# Comparison of log file‐based and measurement‐based QA for detecting MLC positional errors and evaluating dosimetric impacts of MLC defects

**DOI:** 10.1002/acm2.70389

**Published:** 2025-11-18

**Authors:** Chul Hang Kim, Ki Mun Kang, Hoon Sik Choi

**Affiliations:** ^1^ Department of Radiation Oncology Gyeongsang National University School of Medicine and Gyeongsang National University Changwon Hospital Changwon Republic of Korea; ^2^ Department of Biomedical Engineering Pusan National University Pusan Republic of Korea; ^3^ Institute of Health Science Gyeongsang National University Jinju Republic of Korea

**Keywords:** log file‐based QA, measurement‐based QA, mechanical wear, MLC positional accuracy

## Abstract

**Purpose:**

This study aimed to compare the sensitivity of log file‐based quality assurance (LF‐QA) and measurement‐based quality assurance (MB‐QA) for detecting multileaf collimator (MLC) positional errors and to evaluate the dosimetric impacts of MLC mechanical drive train defects.

**Methods:**

Mechanical degradation of the MLC was simulated on a TrueBeam STx system by inducing three defect types: T‐nut surface wear (0.5–1.2 mm), drive screw thread wear, and motor degradation. MLC positioning accuracy was assessed using a rotational Picket Fence (PF) test, and the dosimetric impacts were evaluated on clinical breast intensity‑modulated radiation therapy (IMRT) and prostate volumetric‑modulated arc therapy (VMAT) plans. LF‐QA and MB‐QA were performed concurrently under identical delivery conditions. Gamma passing rates (GPRs) and dose‐volume histogram (DVH) analyses were compared between baseline and defective deliveries.

**Results:**

LF‐QA detected positional deviations between baseline and defective conditions (<0.14 mm; *p* < 0.05) but consistently underestimated the extent of the induced defects. Correspondingly, LF‐QA gamma analysis (GPRs ≈ 100%) and DVH metrics (∆*D* < 0.2%) showed no detectable dosimetric differences. MB‐QA exhibited higher sensitivity to specific MLC defects, identifying localized fluence variations for T‐nut surface wear, whereas no discernible differences were observed for drive screw thread wear or motor degradation. MB‐QA gamma analysis revealed localized dose differences of up to 15% in breast IMRT and 7.4% in prostate VMAT. DVH analysis further demonstrated clinically relevant dose variations in organs at risk (OARs), including the contralateral breast (Δ*D*_mean_: 5.52%) and right lung (Δ*D*_1_: 4.50%) in breast IMRT, and the penile bulb (Δ*D*_99_: 1.55%) in prostate VMAT.

**Conclusion:**

LF‐QA showed limited sensitivity to sub‐millimeter MLC errors and did not capture clinically meaningful dosimetric deviations under mechanically degraded conditions. MB‐QA enabled superior error detection and clinically relevant dosimetric verification. These findings indicate that LF‐QA alone may be insufficient for patient‐specific QA and that incorporating MB‐QA is essential for ensuring reliable dosimetric verification.

## 1 INTRODUCTION

High‐precision radiotherapy techniques, such as intensity‑modulated radiation therapy (IMRT) and volumetric‑modulated arc therapy (VMAT), enable the delivery of highly conformal dose distributions within sub‐centimeter target margins, thereby minimizing irradiation of surrounding organs at risk (OARs). The accuracy of these techniques critically depends on the mechanical performance of the multileaf collimator (MLC), which dynamically modulates beam intensity during treatment delivery.^1^, ^2^ With prolonged clinical operation, cumulative mechanical wear can occur in MLC components, such as T‐nuts, drive screws, and motors, potentially leading to subtle positional deviations that can compromise dosimetric accuracy.^3^

Previous studies have shown that sub‐millimeter MLC positional errors can result in dosimetric variations in both breast IMRT and prostate VMAT plans. Li et al. (2025)^4^ demonstrated that MLC leaf positioning errors in breast IMRT plans significantly affect QA metrics and can lead to noticeable dose variations, while Oliver et al. (2011)^5^ and Betzel et al. (2012)^6^ found about a 2% reduction in target dose for prostate VMAT plan under comparable conditions. Moreover, several reports have indicated that the dosimetric sensitivity to such errors increases with plan complexity, as highly modulated plans tend to amplify small geometric deviations.^7^, ^8^ In stereotactic body radiation therapy (SBRT), where sub‐millimeter geometric precision is essential, even small deviations may compromise local tumor control and increase the risk of toxicity, such as radiation pneumonitis.^9^, ^10^, ^11^ Accordingly, the American Association of Physicists in Medicine (AAPM) Task Group 198 and Medical Physics Practice Guideline 8.b recommend periodic verification of MLC performance to ensure clinically acceptable accuracy. TG‐198 specifies a tolerance of ±1 mm for MLC leaf positioning in IMRT, whereas MPPG 8.b requires an accuracy within 0.5 mm for clinically relevant leaf positions, which serves as the action limit.^12^, ^13^

Conventional measurement‐based quality assurance (MB‐QA) methods, including electronic portal imaging devices (EPIDs), film dosimetry, and diode arrays, are widely used to verify MLC positioning and ensure dosimetric fidelity. Although these methods provide high accuracy, their considerable time and labor demands pose challenges for routine use across all treatment plans. Moreover, as treatment planning becomes more efficient and the number of patients receiving advanced radiotherapy techniques continues to increase, such measurement‐based verification may result in a continued increase in clinical workload.^14^, ^15^ In contrast, log file‐based QA (LF‐QA) offers an automated, time‐efficient workflow by recording delivery parameters such as leaf positions, gantry angles, and monitor units with high temporal resolution.^16^, ^17^, ^18^, ^19^, ^20^ However, because LF‐QA relies on encoder‐derived leaf positions rather than direct measurements, it has reduced sensitivity to certain mechanical deviations.^21^, ^22^, ^23^, ^24^ Barnes et al. (2022)^24^ highlighted this limitation by demonstrating the insensitivity of LF‐QA to T‐nut‐induced backlash, underscoring the need for a more comprehensive evaluation across different defect types and delivery systems.

Building upon this context, the present study aimed to provide a systematic evaluation of MLC performance degradation by (1) experimentally inducing clinically relevant defects, including T‐nut surface wear similar to that of Barnes et al. (2022),^24^ but additionally driving screw thread wear and motor performance degradation; (2) directly comparing the detection sensitivity of LF‐QA and MB‐QA using a high‐definition 120 (HD120) MLC system; and (3) extending the evaluation beyond positional accuracy to assess the dosimetric consequences for patient‐specific treatment plans through gamma index and dose‐volume histogram (DVH) analyses.

## 2 MATERIALS AND METHODS

### 2.1 Treatment delivery system

All experiments were conducted using a TrueBeam STx linear accelerator (Varian Medical Systems, Palo Alto, CA, USA; installed 2016; v.2.0) equipped with an HD120 MLC. The MLC comprises 120 tungsten alloy leaves arranged in two opposing banks; each bank consists of 32 central leaves (2.5 mm width) and 28 peripheral leaves (5.0 mm width). Each leaf is coupled to a drive screw via a T‐nut, which provides the mechanical interface between the components and reduces the risk of damage to the motor and drive assembly in the event of a collision. Leaf position is continuously monitored by a dual‐feedback system consisting of primary motor encoders and secondary potentiometers (Softpot), enabling independent positional verification.

### 2.2 Induced mechanical defects

To evaluate the detection sensitivity of each QA method, three types of mechanical defects were deliberately induced in the MLC system: T‐nut surface wear, drive screw thread wear, and motor performance degradation. The specific MLC leaves designated for each defect type are summarized in Table 1.

**TABLE 1:** Manually induced mechanical defects in multileaf collimator (MLC) leaves.

Defect type	Leaf no.	Evaluation method	Degree of wear
T‐nut surface wear	A08, B48	Vernier caliper	0.58 ± 0.03 mm
	A24, B30		0.81 ± 0.03 mm
	A50, B12		0.98 ± 0.05 mm
	A32, B10		1.22 ± 0.04 mm
Drive screw thread wear	A06, B34	Backlash test	0.45 ± 0.08 mm
Motor degradation	A10, B16	PWM test	18.06 ± 1.51%

*Note*: To facilitate T‐nut replacement, six even‐numbered leaves were selected from each carriage. Adjacent leaf pairs were deliberately avoided to minimize inter‐leaf interference. The degree of wear is represented by the mean ± standard deviation derived from three repeated measurements using the corresponding evaluation methods. Abbreviations: PWM, pulse width modulation.

#### 2.2.1 T‐nut surface wear

**FIGURE 1:**
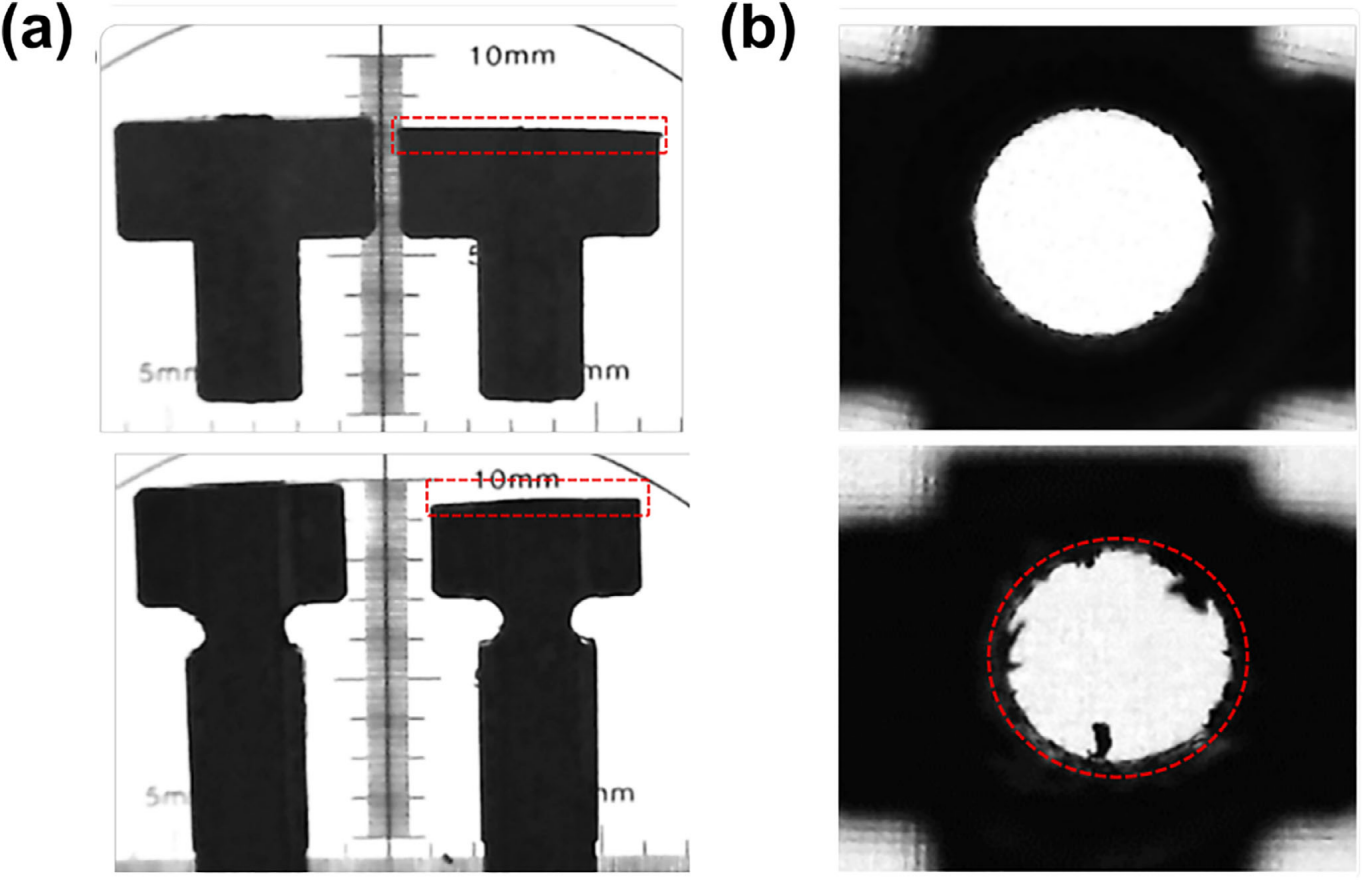
Scanning electron microscopy (SEM) images of mechanical wear in MLC T‐nuts: (a) external surfaces of quarter‐width (top) and half‐width (bottom) leaf T‐nuts, illustrating worn (right) and non‐worn (left) surfaces. (b) Internal thread structures of T‐nuts, contrasting intact (top) with worn (bottom) components. Dashed red outlines indicate worn regions. MLC, multileaf collimator.

To represent sub‐millimeter mechanical degradation and to evaluate the minimum wear detectable by each QA method, four T‐nuts from each MLC bank were abraded with 180‐ and 320‐grit sandpaper to wear levels of 0.5, 0.8, 1.0, and 1.2 mm. Wear depths were periodically measured throughout the abrasion process using a digital Vernier caliper (Mitutoyo 530‐181, Japan). To account for surface non‐uniformity resulting from manual abrasion, measurements were performed at four different points on each T‐nut surface. Scanning electron microscopy (SEM) was used to compare unworn and worn T‐nuts and to visualize the extent of surface wear (Figure 1a).

#### 2.2.2 Drive screw thread wear

To simulate thread wear, a spare drive screw was mounted on a variable‐speed drill and inserted into two selected T‐nuts, then repeatedly rotated in both directions to abrade the internal threads. The extent of drive screw thread wear was evaluated using a backlash test in the system service mode, which estimates the mechanical clearance from motor‐count differences during directional changes, yielding values of 0.18 ± 0.06 mm for unworn T‐nuts and 0.46 ± 0.13 mm for worn T‐nuts. SEM images of unworn and worn threads are shown in Figure 1b.

#### 2.2.3 Motor performance degradation

To simulate motor performance degradation, two leaf motors previously retired from clinical service were utilized. The performance of these units was quantitatively characterized via pulse width modulation (PWM) measurements acquired in the system service mode (four acquisitions per motor). The retired units were confirmed to be degraded, with a mean PWM of 18.1 ± 3.4% and maximum values exceeding the manufacturer's operational limit of 20.0%. In contrast, clinically functional motors demonstrated a lower mean PWM of 10.7 ± 0.6%, with maximum values not exceeding 14.0%.

### 2.3 Evaluation of MLC positional accuracy

MLC positional accuracy was evaluated under both baseline (non‐defective) and defect‐induced conditions using a rotational Picket Fence (PF) test plan obtained from the Varian support portal.^25^ The plan delivered 10 sequential radiation strips during a continuous gantry rotation from 179° to 181° (IEC 61217 standard), during which trajectory log files and EPID images were concurrently acquired for each condition.

Data processing and analysis were performed using an in‐house software developed in LabVIEW (National Instruments, Austin, TX, USA). For the LF‐QA, positional deviations were quantified by extracting leaf positions at each control point and comparing them between baseline and defect‐induced conditions. For the MB‐QA, the acquired EPID images (1190 × 1190 pixels) were analyzed. Within the region of interest (ROI), pixel data were normalized, and transverse and longitudinal profiles were extracted at the positions of defect‐induced leaves. Longitudinal profiles were used to assess adjacent leaf alignment. Transverse profiles were analyzed with the FWHM method, and linear interpolation at the half‐maximum points was applied to measure opposing leaf gaps with sub‐pixel accuracy.

### 2.4 Dosimetric impact assessment of MLC mechanical defects

The study was conducted under limited machine availability and focused on breast and prostate treatment plans, which represent typical clinical sites for IMRT and VMAT, respectively. A total of 15 clinical radiotherapy plans (8 left‐breast IMRT and 7 prostate VMAT cases) were analyzed. These plans were designed with uniform geometries and optimization parameters, enabling reliable and reproducible assessment of MLC error detection sensitivity. This evaluation was conducted with the approval and review of the Institutional Review Board.

#### 2.4.1 Two‐dimensional gamma index analysis

Log files and EPID images were acquired under both baseline and defect‐induced conditions. Each log file was processed using an in‐house Python script to extract leaf positions, gantry angles, and monitor units and converted into the Digital Imaging and Communications in Medicine Radiation Therapy (DICOM‐RT) format. The log‐derived DICOM files were imported into the treatment planning system (TPS; Eclipse v13.7, Varian Medical Systems, Palo Alto, CA, USA) to recalculate portal dose distributions using the Portal Dose Image Prediction (PDIP) algorithm.

For each treatment plan, portal dose distributions obtained under baseline and defect‐induced conditions, from both log files and EPID measurements, were compared using the Portal Dosimetry Software (Varian Medical Systems). Gamma index analysis was performed to compare baseline and defect‐induced conditions with a 2%/2 mm criterion. The corresponding gamma passing rates (GPRs) were evaluated using the Wilcoxon signed‐rank test in SPSS (v24, IBM Corp., Armonk, NY, USA), as the Shapiro–Wilk test indicated that the data did not satisfy normality (*p* < 0.05). A significance threshold of *p* < 0.05 was applied for all comparisons.

#### 2.4.2 Three‐dimensional dose‐volume histogram (DVH) analysis

Log file‐based volume dose distributions were calculated in the TPS from DICOM‐RT files (Section 2.4.1) using the Anisotropic Analytical Algorithm (AAA). In parallel, dose measurements were acquired with the MatriXX Evolution 2D ionization chamber array (IBA Dosimetry, Schwarzenbruck, Germany), and three‐dimensional dose distributions were calculated in COMPASS software (IBA Dosimetry, Schwarzenbruck, Germany).

DVH metrics were used to compare baseline and defect‐induced conditions. For breast IMRT plans, the volume of interest (VOIs) included the clinical target volume (CTV), left lung, right lung, heart, contralateral breast, and spinal cord. For prostate VMAT plans, the VOIs included PTV_7000_, PTV_6600_, PTV_4500_, bladder, rectum, bowel, anal canal, penile bulb, and bilateral femoral heads. For each VOI, D_99_ (dose received by 99% of the volume), *D*_mean_ (mean dose), and *D*_1_ (dose to the hottest 1% of the volume) were analyzed.

The relative dose deviation (∆*D*) for each metric was calculated as follows:

$$\Delta D_{M}\left(\%\right)=\frac{D_{M,\mathrm{defect}}-D_{M,\mathrm{baseline}}}{D_{M,\mathrm{baseline}}}\times 100$$

where *D* denotes the dose metric (*D*_99_, *D*_mean_, or *D*_1_) and M the QA method (LF‐QA or MB‐QA). *D*_M,defect_ and *D*_M,baseline_ represent values under defect‐induced and baseline conditions, respectively. Wilcoxon signed‐rank tests were used to evaluate statistical significance (*p* < 0.05).

## 3 RESULTS

### 3.1 Sensitivity of QA systems to MLC mechanical defects

**FIGURE 2:**
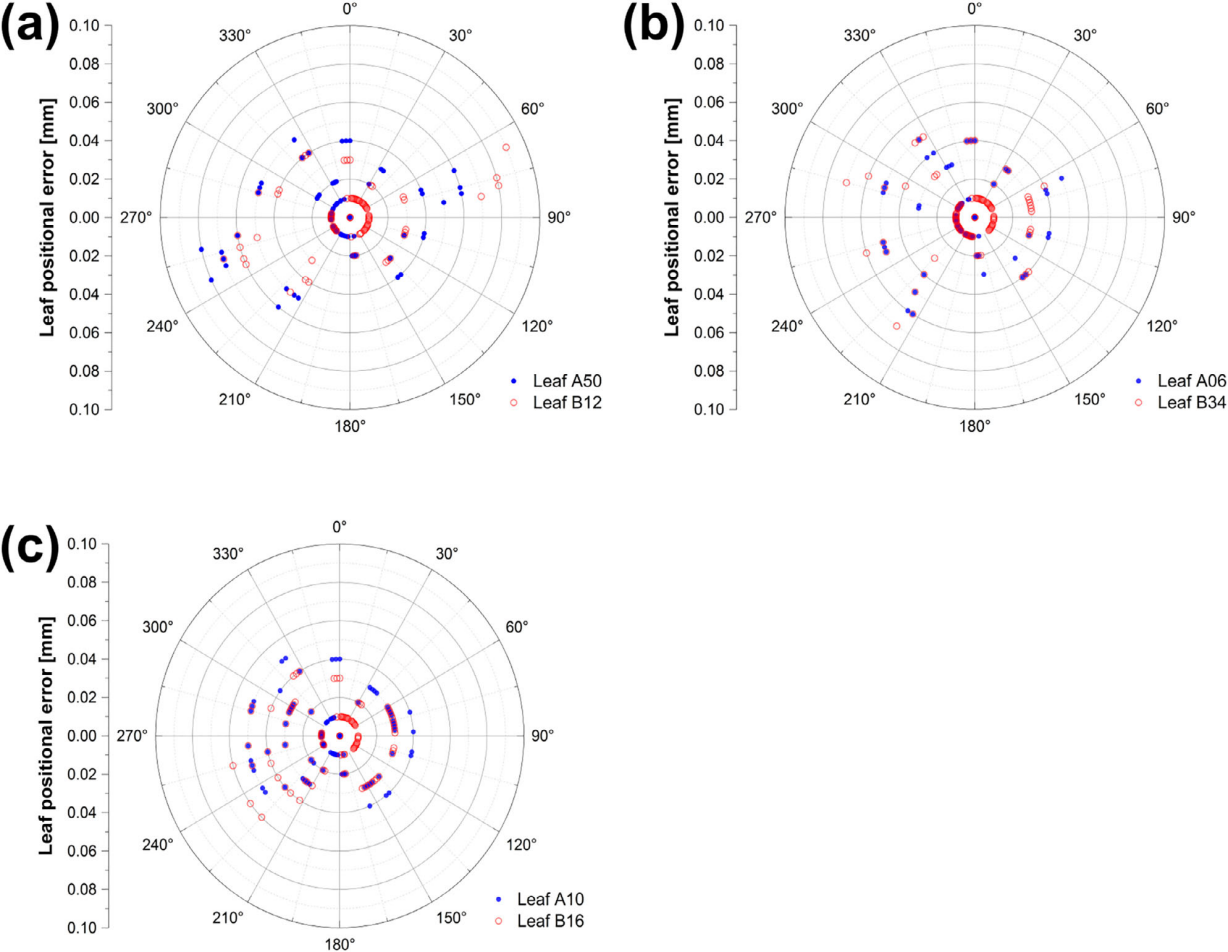
Leaf‐by‐leaf positional errors extracted from log files and plotted as a function of gantry angle for each type of mechanical defect: (a) surface wear of T‐nuts, (b) internal thread wear in T‐nuts, and (c) motor performance degradation.

**FIGURE 3:**
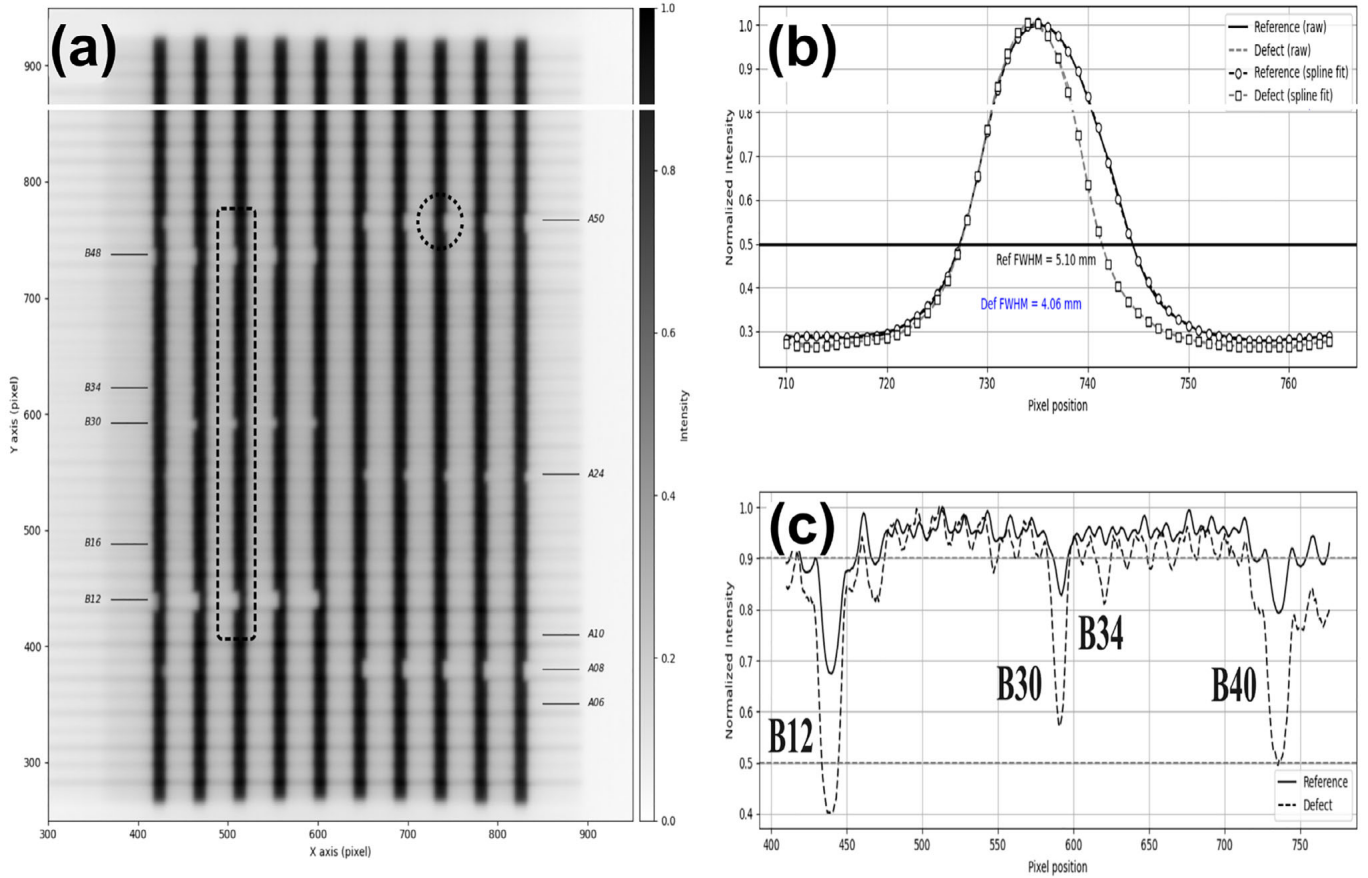
Rotational Picket Fence (PF) test using EPID imaging to assess MLC positional accuracy during gantry rotation: (a) PF image acquired during gantry rotation, (b) intensity profiles used for FWHM analysis, and (c) normalized profiles showing localized reductions at defective MLC leaves (B12, B30, B34, and B40). EPID, electronic portal imaging device; MLC, multileaf collimator.

LF‐QA detected positional deviations for all induced defects (<0.14 mm; *p* < 0.05), but consistently underestimated the physically introduced wear depths (Table 2). The maximum reported deviations were 0.14 mm for T‐nut surface wear, 0.07 mm for drive screw thread wear, and 0.06 mm for motor degradation (Figure 2). For T‐nut surface wear, defects were induced in eight leaves; however, two leaves were excluded from EPID analysis due to repeated system interlocks during beam delivery, leaving six leaves for evaluation. In the EPID‐based PF analysis, discernible fluence variations were evident for T‐nut surface wear, but not for drive screw thread wear or motor degradation. Detection of T‐nut surface wear was dependent on gantry angle, with the largest differences when leaf travel was parallel to the gravitational vector (Figure 3). FWHM analysis of EPID images closely approximated the 1.0 mm reference error but underestimated deviations smaller than 1.0 mm and those involving narrow 2.5‐mm leaves (Table 2).

**TABLE 2:** Maximum leaf positional errors measured using log files and EPID under induced mechanical defects.

Defect type	Degree of wear	Log file (mm)	EPID (mm)
T‐nut surface wear	0.58 ± 0.03 mm	0.05	0.48
	0.81 ± 0.03 mm	0.08	0.86
	0.98 ± 0.05 mm	0.09	1.04
	1.22 ± 0.04 mm	0.14	N/A
Drive screw thread wear	0.45 ± 0.08 mm	0.07	0.34
Motor degradation	18.06 ± 1.51%	0.06	0.03

*Note*: N/A = Measurement was not possible due to system interlock activation.

Abbreviations: EPID, electronic portal imaging device; N/A, not applicable.

### 3.2 Dosimetric impact of MLC mechanical defects

#### 3.2.1 Two‐dimensional gamma index analysis

**FIGURE 4:**
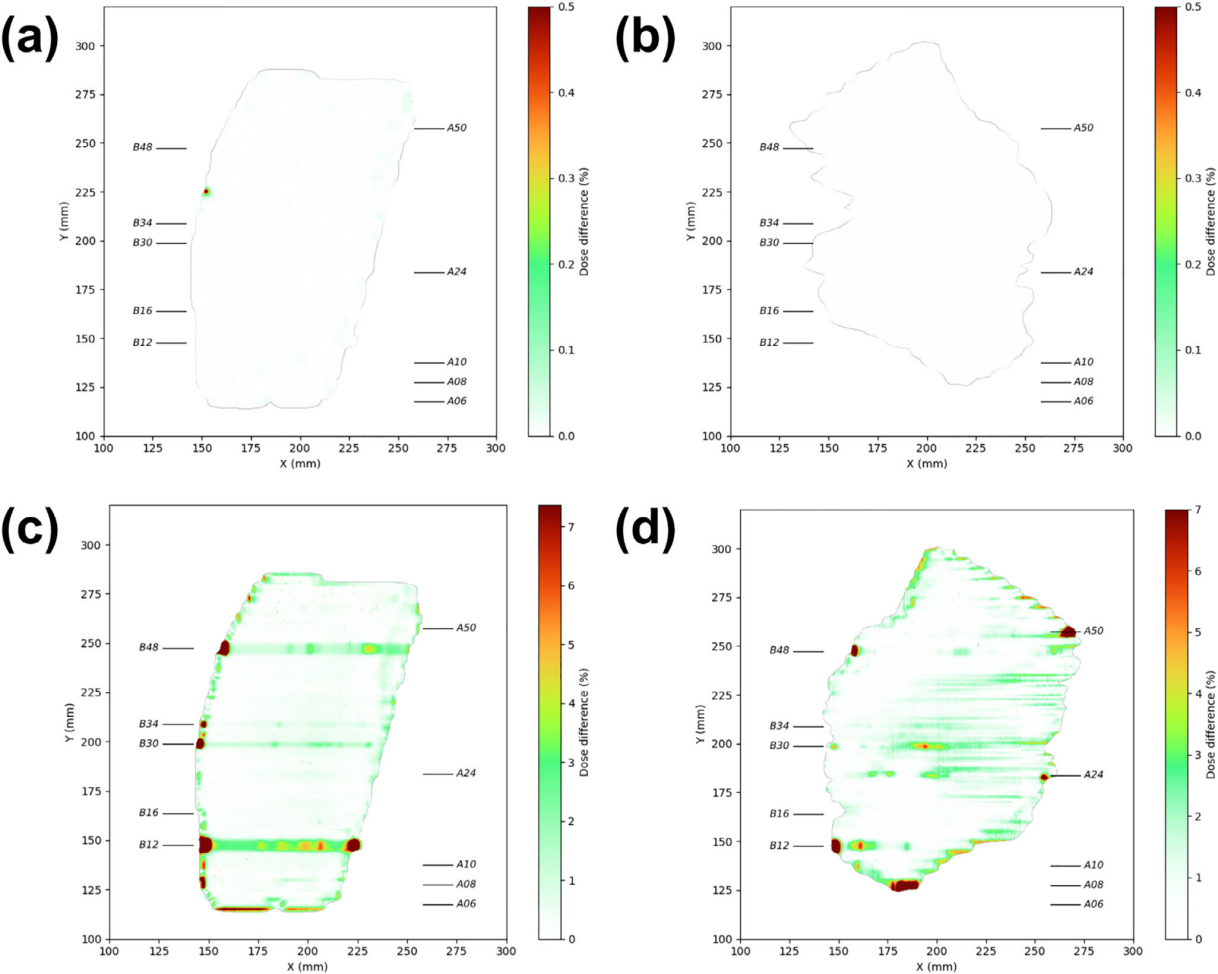
Two‐dimensional dose distributions reconstructed from log files and EPID measurements, comparing deliveries with and without MLC mechanical defects: (a) breast IMRT (log file‐based), (b) prostate VMAT (log file‐based), (c) breast IMRT (EPID‐based), and (d) prostate VMAT (EPID‐based). EPID, electronic portal imaging device; IMRT, intensity‑modulated radiation therapy; MLC, multileaf collimator; VMAT, volumetric‑modulated arc therapy.

Figure 4 compares the 2D dose distributions for representative breast IMRT and prostate VMAT fields, reconstructed from log files and measured by the EPID. The recalculated dose distributions from LF‐QA showed no discernible differences from the baseline plans (Figure 4a,b). In contrast, EPID measurements revealed localized dose differences of up to 8% for the breast IMRT plan and 5% for the prostate VMAT plan in the same fields, predominantly at the field edges where steep dose gradients were present (Figure 4c,d).

Gamma analysis using a 2%/2 mm criterion quantified these observations. The LF‐QA yielded GPRs of 100% for both IMRT and VMAT plans, with maximum local dose deviations below 0.2%. In contrast, the EPID‐based QA yielded mean GPRs of 98.4% ± 1.3% for IMRT and 98.9% ± 0.7% for VMAT, with maximum local dose deviations of 15% and 7.4%, respectively.

#### 3.2.2 DVH‐based dosimetric analysis

DVH analysis based on log file‐derived 3D reconstruction showed minimal changes across all structures (Δ*D* < 0.2%) for both IMRT and VMAT plans, without statistically significant differences (*p* > 0.05).

**FIGURE 5:**
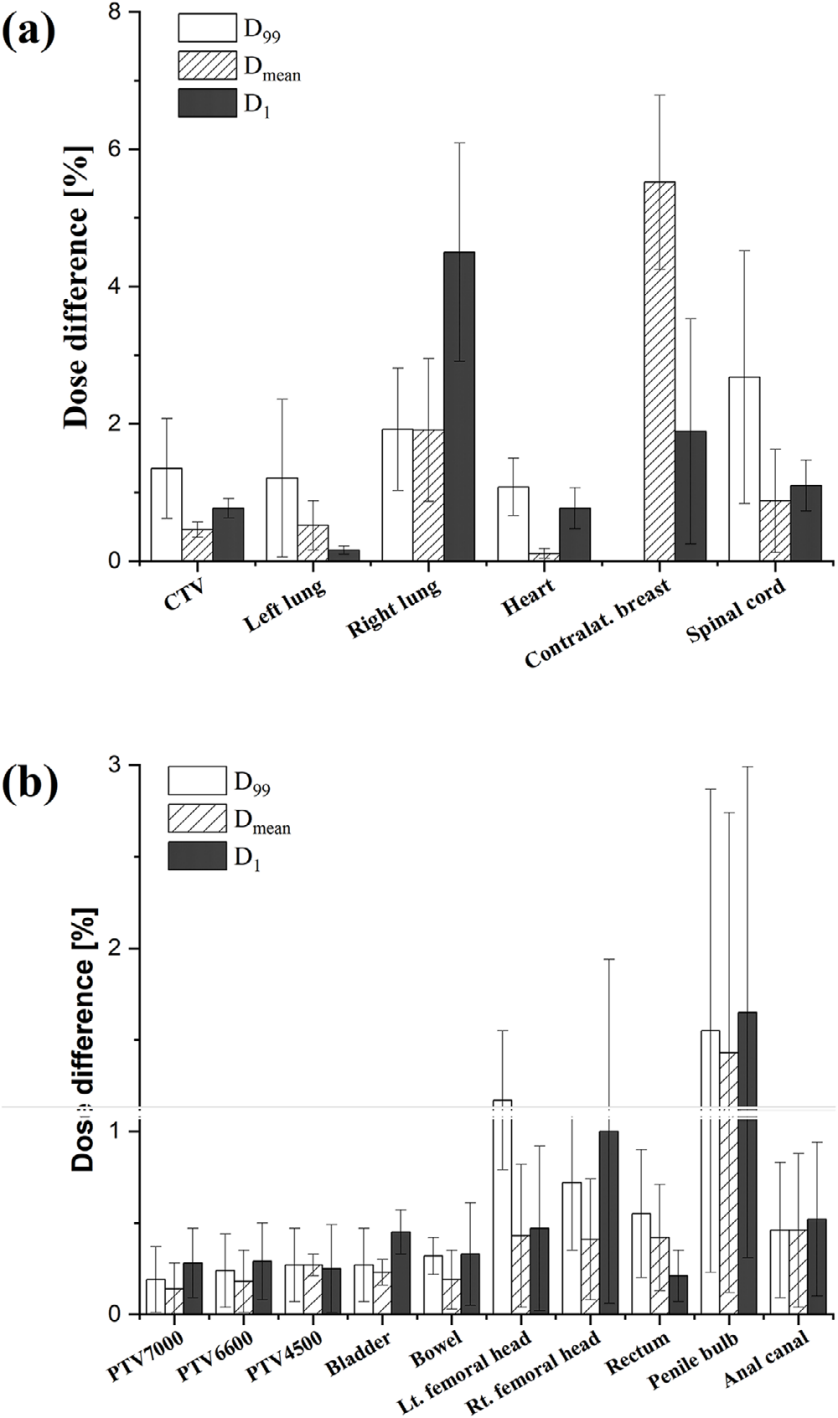
Absolute percentage differences in *D*_99_, *D*_mean_, and *D*_1_ between baseline and conditions with defects. Note: Data were reconstructed employing the COMPASS system, utilizing MatriXX evolution measurements. Error bars indicate one standard deviation.

In contrast, DVH analysis using 3D dose distributions reconstructed from MatriXX detector measurements revealed dose deviations in both plans (Figure 5). For the breast IMRT plans, the largest dose changes were observed in the contralateral breast (Δ*D*_mean_: 5.52% ± 1.27%) and the right lung (Δ*D*_1_: 4.50% ± 1.59%). Dose changes were also observed in the CTV (Δ*D*_99_: 1.35% ± 0.73%) and the spinal cord (Δ*D*_99_: 2.68% ± 1.84%). Conversely, changes in the heart (Δ*D*_1_: 0.77% ± 0.30%) and the left lung (Δ*D*_1_: 0.16% ± 0.06%) were smaller. For the prostate VMAT plans, dose variations in all target volumes (ΔPTV_7000_, ΔPTV_6600_, and ΔPTV_4500_) remained below 0.4% for most DVH metrics. For the OARs, the penile bulb showed the most notable dose variations, with mean changes of 1.55% ± 1.32% (Δ*D*_99_), 1.65% ± 1.34% (Δ*D*_1_) and 1.43% ± 1.31% (Δ*D*_mean_). The rectum exhibited a mean variation of 0.42% ± 0.29% (Δ*D*_mean_), and the bilateral femoral heads showed variations of 0.43% ± 0.39% (left) and 0.41% ± 0.33% (right). Dose variations in the remaining OARs, including the anal canal, bladder, and bowel, were all below 0.6%.

## 4 DISCUSSION

This study showed a clear disparity between LF‐QA and MB‐QA in detecting mechanical MLC defects and assessing their dosimetric impact. In agreement with Barnes et al. (2022),^24^ a comparable difference between the two QA approaches was observed under T‐nut wear, confirming the limited sensitivity of LF‐QA relative to EPID‐based verification. Building upon these findings, the present study further investigated additional defect types and quantified their dosimetric consequences in clinically relevant treatment plans. While LF‐QA detected positional differences between baseline and defective conditions, it consistently underestimated the magnitude of leaf deviations. This limitation arises from its reliance on motor encoder data, which cannot account for mechanical failures such as backlash or thread wear, as demonstrated in prior studies.^22^, ^23^, ^24^

Accordingly, LF‐QA may overlook clinically significant dosimetric errors, potentially leading to inaccurate assessment of treatment delivery accuracy.

In contrast, MB‐QA using EPID measurements demonstrated higher sensitivity for certain defect types. Consistent with previous reports,^24^, ^26^ portal dosimetry revealed localized fluence variations for leaves affected by T‐nut surface wear, particularly when leaf motion was aligned with gravity, indicating gantry‐angle‐dependent effects on MLC performance. By contrast, drive screw thread wear resulted in minimal or undetectable MLC positional errors. This can be explained by non‐uniform thread wear in combination with leaf motion direction and gantry angle, which led to substantial variations in individual leaf positioning errors. In addition, the mechanical impact of drive screw thread wear on leaf positioning was either negligible or below the detector's spatial resolution threshold, making such wear‐induced deviations difficult to detect. Under motor degradation, an increase in PWM value may indicate higher mechanical load or transient strain on the motor but did not translate into measurable MLC positional errors. Accordingly, no discernible variations were observed in the fluence distribution. These findings suggest that MB‐QA sensitivity varies with the detector's spatial resolution, the magnitude and reproducibility of mechanical deviations, and the interaction between defect types and delivery geometry.

Statistically significant differences between baseline and defect‐induced conditions were confirmed in the PF test and gamma index analysis. By contrast, although variations were observed in the COMPASS‐based DVH analysis, these differences did not reach statistical significance in the Wilcoxon signed‐rank test. This lack of statistical significance may reflect not only the limited sample size and inter‐patient variability, but also the limited sensitivity of DVH metrics to localized dose variations near MLC defect regions, since DVH represents a cumulative dose‐volume relationship that integrates dose contributions over each evaluated structure.

In addition, the FWHM analysis tended to underestimate displacements for narrow leaves (2.5 mm width) and sub‐millimeter errors. As reported in previous studies,^26^, ^27^, ^28^ this underestimation is considered to be attributed to the radiological leaf‐width narrowing inherent to the HD120 MLC design^26^ and the limited spatial sampling and interpolation accuracy of EPID‐based measurements.^27^, ^28^ Gamma index and DVH metrics derived from log files showed negligible differences between baseline and defect conditions. Minor dose deviations were observed in regions unrelated to the induced MLC defects, likely arising from differences in the sampling rates of independently acquired log files. Conversely, MB‐QA clearly identified dose discrepancies along the trajectories of defective leaves, especially in high‐dose regions and at steep dose gradients. These observations are consistent with previous reports that even sub‐millimeter MLC errors can lead to clinically significant dosimetric deviations.^22^, ^23^, ^24^

The dosimetric impact of MLC errors was more pronounced in IMRT than in VMAT plans. This is likely because fixed‐gantry IMRT concentrates delivery errors within localized regions.^6^ In our breast IMRT plans, the combination of a fixed collimator angle (0°), unidirectional leaf motion, and restricted gantry angles (300°, 315°, 115°, and 120°) designed to spare the heart and lungs amplified the dosimetric impact, leading to substantial dose deviations in the ipsilateral lung and contralateral breast. In contrast, prostate VMAT employed two arcs with different collimator angles (30° and 330°) and bidirectional leaf motion, which distributed the delivery errors across the arc trajectory and thereby mitigated localized dosimetric consequences.

Collectively, these findings indicate that LF‐QA alone may be insufficient for machine QA and patient‐specific quality assurance (PSQA). Although LF‐QA detects small positional differences, it does not reliably translate them into patient‐specific dosimetric consequences. MB‐QA confirmed that subtle defects can compromise OAR sparing and target coverage. In conjunction with prior TPS‐based simulation studies,^29^, ^30^, ^31^, ^32^ this work provides complementary empirical evidence by experimentally inducing mechanical defects and assessing their clinical impact, including the dosimetric consequences of sub‐millimeter MLC errors. Zwan et al. (2016)^33^ also analyzed the dosimetric effects of simulated MLC positional errors in VMAT and found that DVH metric changes were minimal for sub‐millimeter deviations. In contrast, the present study observed noticeable changes in both gamma analysis and DVH metrics for sub‐millimeter MLC positional errors that occurred under actual mechanical degradation, indicating that mechanical degradation in clinical systems can lead to more pronounced dosimetric variations than those predicted by simulations.

This study has several limitations. First, drive‐screw thread wear could not be measured directly due to physical access constraints and was instead inferred from backlash tests and SEM observations. Second, the PF test used unidirectional leaf motion, which may not fully represent the bidirectional behavior typical of clinical deliveries. Third, the dosimetric impact likely varies with anatomical site, treatment complexity, and defect severity. Finally, the study was conducted on a single linac platform; validation across additional systems and institutions is warranted.

## 5 CONCLUSION

This study demonstrated that LF‐QA has limited sensitivity to sub‐millimeter MLC errors and does not reliably capture their clinically relevant dosimetric consequences under mechanically degraded conditions. In contrast, MB‐QA showed higher sensitivity to specific MLC defects and detected dose variations of clinical significance. While LF‐QA remains useful as a complementary tool for long‐term monitoring of MLC performance and system integrity, our findings suggest that exclusive reliance on LF‐QA for PSQA may be insufficient. Accordingly, incorporating MB‐QA into PSQA is recommended to enhance the accuracy and safety of treatment delivery.

## AUTHOR CONTRIBUTIONS

Chul Hang Kim contributed to the study design, experimental work, and manuscript writing. Ki Mun Kang contributed to the study design and clinical interpretation of results. Hoon Sik Choi contributed to the study design, data discussion, and manuscript writing.

## CONFLICT OF INTEREST STATEMENT

The authors declare no conflict of interest.

## ETHICS APPROVAL

This study was approved by the Institutional Review Board of Gyeongsang National University Changwon Hospital (IRB No. GNUCH‐NON2025‐003).
